# Predominant HLA Alleles and Haplotypes in Mild Adverse Drug Reactions Caused by Allopurinol in Vietnamese Patients with Gout

**DOI:** 10.3390/diagnostics11091611

**Published:** 2021-09-03

**Authors:** Chu Van Son, Nguyen Thi Hong Loan, Tran Huyen Trang, Le Xuan Thinh, Nguyen Ba Khanh, Le Thi Hong Nhung, Nguyen Van Hung, Tran Ngoc Que, Nguyen Van Lieu, Pham Dinh Tung, Nguyen Thi Van Anh, Nguyen Dinh Thang

**Affiliations:** 1 Key Laboratory of Enzyme and Protein Technology, VNU University of Science, Vietnam National University-Hanoi, 334 Nguyen Trai, Thanh Xuan, Hanoi 120017, Vietnam; sonssc238@gmail.com (C.V.S.); loannguyen@hus.edu.vn (N.T.H.L.); nhungle@hus.edu.vn (L.T.H.N.); 2 Department of Internal Medicine, Hanoi Medical University, 1 Ton That Tung, Dong Da, Hanoi 116001, Vietnam; tranhuyentrang@hmu.edu.vn (T.H.T.); hungnguyen@hmu.edu.vn (N.V.H.); 3 Department of Rheumatology, Bach Mai Hospital, 78 Giai Phong, Phuong Mai, Dong Da, Hanoi 116305, Vietnam; thinhhuyethoc@gmail.com (L.X.T.); khanhhhtm@gmail.com (N.B.K.); 4 Stem Cell Bank, National Institute of Hematology and Blood Transfusion, Pham Van Bach, Cau Giay, Hanoi 122000, Vietnam; drque72@gmail.com; 5 Department of Neuroscience, Tam Anh General Hospital, 108 Hoang Nhu Tiep, Bo De, Long Bien, Hanoi 125300, Vietnam; lieutk@gmail.com; 6 Department of Probability and Statistics, Faculty of Mathematics-Mechanics-Informatics, VNU University of Science, Vietnam National University, 334 Nguyen Trai, Thanh Xuan, Hanoi 120017, Vietnam; tungphd85@gmail.com

**Keywords:** gout, allopurinol, mild cutaneous adverse drug reactions (MCARs), HLA-B^*^58:01 allele, one-locus haplotypes, HLA-A^*^02:01/HLA-A^*^24:02, HLA-A^*^02:01/HLA-A^*^29:01

## Abstract

Allopurinol (ALP) is commonly used as a drug for gout treatment. However, ALP is known to cause cutaneous adverse reactions (CARs) in patients. The HLA-B^*^58:01 allele is considered a biomarker of severe CAR (SCAR) in patients with gout, with symptoms of Stevens Johnson syndrome, and with toxic epidermal necrolysis. However, in patients with gout and mild cutaneous adverse drug reactions (MCARs), the role of HLA-allele polymorphisms has not been thoroughly investigated. In this study, 50 samples from ALP-tolerant patients and ALP-induced MCARs patients were genotyped in order to examine the polymorphisms of their HLA-A and HLA-B alleles. Our results showed that the frequencies of HLA-A^*^02:01/HLA-A^*^24:02 and HLA-A^*^02:01/HLA-A^*^29:01, the dual haplotypes in HLA-A, in patients with ALP-induced MCARs were relatively high, at 33.3% (7/21), which was HLA-B^*^58:01-independent, while the frequency of these dual haplotypes in the HLA-A locus in ALP-tolerant patients was only 3.45% (1/29). The HLA-B^*^58:01 allele was detected in 38% (8/21) of patients with ALP-induced MCARs, and in 3.45% (1/29) of ALP-tolerant patients. Notably, although HLA-B^*^58:01 may be a cause for the occurrence of MCARs in patients with gout, this correlation was not as strong as that previously reported in patients with SCAR. In conclusion, in addition to the HLA-B^*^58:01 allele, the presence of the dual haplotypes of HLA-A^*^02:01/HLA-A^*^24:02 and/or HLA-A^*^02:01/HLA-A^*^29:01 in the HLA-A locus may also play an important role in the appearance of ALP-induced MCARs in the Vietnamese population. The obtained primary data may contribute to the development of suitable treatments for patients with gout not only in Vietnam but also in other Asian countries.

## 1. Introduction

Allopurinol (ALP) is an analog of purine hypoxanthine, and is commonly prescribed for the treatment of hyperuricemia and gout. In general, ALP can decrease the level of uric acid by inhibiting xanthine oxidase, which plays an important role in converting xanthine and hypoxanthine to uric acid. In the liver, allopurinol is easily oxidized to oxypurinol, a xanthine oxidase inhibitor [1,2,3,4].

ALP may cause hypersensitivity in patients with gout. It is the most common cause of severe cutaneous adverse reactions (SCARs), including Stevens Johnson syndrome (SJS), toxic epidermal necrolysis (TEN), drug reaction with eosinophilia and systemic symptoms (DRESS), and mild cutaneous adverse reactions (MCARs) [5]. SJS and TEN are life-threatening conditions associated with fever, hepatitis, an increased number of white blood cells, acute renal failure and mild symptoms, and are a cause of pain, itchiness and unpleasantness for patients [1,6].

To date, although the mechanism behind HLA-B^*^58:01-specific ALP-induced adverse drug reactions still remains unclear, it is proposed that allopurinol—or its metabolite, oxypurinol—may be incorporated with the self-peptides of the target cell to form a hapten; then, this hapten will undergo antigen processing before being presented specifically by HLA-B^*^58:01 to activate T-CD8(+) cells, which trigger a T-cell-mediated hypersensitivity reaction [7,8,9,10]. The frequency of the HLA-B^*^58:01 allele varies among ethnic subpopulations. In particular, the frequencies of the HLA-B^*^58:01 allele in Han Chinese, Taiwanese, Korean, Vietnamese, and European populations is 10–15%, 12%, 6–8%, 7–8%, and 1–2%, respectively [11,12,13,14,15,16,17,18,19,20,21,22]. Previous studies have revealed that the HLA-B^*^58:01 allele is strongly associated with SCARs caused by ALP. The frequency of the HLA-B^*^58:01 allele in patients with ALP-induced SCARs, ALP-tolerant patients, and healthy individuals is approximately 90–100%, 10–15%, and 8–10%, respectively [11,12,13,14,15,16,17,18,19,20,21,22]. 

However, there are a significant number of HLA-B^*^58:01-negative patients with ALP-induced SCARs, which implies that the presence of the HLA-B^*^58:01 allele is not the only requirement, indicating that other variants of HLA or other genetic risk factors may also contribute to the etiology [1,6,19,20,23,24,25]. Furthermore, previous studies have shown a strong relationship between the presence of HLA-B^*^58:01 and severe symptoms such as SJS and TEN in patients with ALP-induced SCARs; however, the relationship between HLA-B^*^58:01 and mild symptoms in patients with gout treated with ALP is unclear [7,8,9,10,11,12,13,14,15,16,17,18]. Therefore, in this study, we aimed to identify novel variants of HLA as genetic markers for ALP-induced MCARs in Vietnamese patients with gout. Although the sample size in this study is limited, these primary data may contribute to clinical decision making suitable for patients with gout in Vietnam, in order to avoid both SCARs and MCARs symptoms, and they indicate the need for further studies on the association of HLA haplotypes with ALP-induced MCARs in Asian countries. 

## 2. Materials and Methods

### 2.1. Control Subjects, Patient Recruitment, Classifications and Sample Collection

A total of 50 patients with gout who were treated with ALP at Bach Mai Hospital and Hanoi Medical University Hospital, Hanoi, Vietnam, were recruited for this study. The study was conducted between July 2020 and March 2021. Among the 50 patients with gout who were treated with ALP, 21 patients exhibited mild symptoms (MCARs). The diagnostic criteria for adverse drug reaction (ADR) symptoms were assessed based on the clinical morphology appearing within three months of the treatment [9]. ALP was regarded as the cause for the appearance of CARs if the SCAR symptoms occurred within the first three months of medication and disappeared after stopping ALP treatment. The criteria for the diagnosis of SJS, SJS/TEN and TEN were set at 10%, 10–30% and >30% of the body’s skin area being detached, respectively. The criteria for DRESS were the occurrences of skin rashes with two or more of the following symptoms: eosinophilia, leukocytosis, atypical circulating lymphocytes, acute hepatocellular injury and worsening renal function. Skin rashes and/or itches not classified as any type of SCAR were considered MCARs. Patients who had no adverse drug reaction were excluded as negative ALP-induced CAR patients [5]. The patients’ blood samples were collected in accordance with accordance with research ethics regulations in medicine. The patients signed consent forms after they were provided with sufficient information on the research and the confidentiality of their personal information.

### 2.2. HLA Genotyping 

The DNA extraction from the blood samples was performed using the QIAamp DNA Blood Mini Kit (Qiagen, Hilden, Germany) with 4 mL serum. The HLA alleles were genotyped for loci A and B by sequence-specific oligonucleotide PCR (SSO-PCR) using the LIFECODES HLA SSO kit (Luminex, Austin, TX, USA) [24]. The HLA allele present in hetero- and/or homogenous forms of the HLA gene was counted as a positive result. The potential ambiguities were diminished by Sanger sequencing [25]. The primer pairs used for the HLA allele genotyping (HLA-A and HLA-B) were obtained from a previous report [26], and are presented as follows: 

Primers for HLA-A alleles:

Ain1-A-M13F 5-TGTAAAACGACGGCCAGTGGGGCGCARGACCCGGGA-3;
Ain1-G-M13F, 5-TGTAAAACGACGGCCAGTGGGCGCAGGACCGGGG-3;
Ain1-T-M13F, 5-TGTAAAACGACGGCCAGTGGGRCGCAGGACCCGGGT-3;
Ain3–62-M13R, 5-CAGGAAACAGCTATGACCGTCCCAATTGTCTCCCCTCCTT-3.

Primers for HLA-B alleles:

Bin1-TA-M13F, 5-TGTAAAACGACGGCCAGTGGCGGGGGCGCAGGACCTGA-3;
Bin1-CGM13F, 5-TGTAAAACGACGGCCAGTCGGGGGCGCAGGACCCGG-3 (F);
Bin3-M13R, 5-CAGGAAACAGCTATGACCGGAGGCCATCCCCGGCGACCTAT-3 (R).

### 2.3. Statistical Analysis 

SPSS software (version 20.0; SPSS Inc., Chicago, IL, USA) was used for the statistical analyses. A Mann-Whitney U test, *t*-test, or Fisher’s exact test was performed to compare the numerical and categorical data between the clinical characteristics of the subjects in the groups (MCARs and tolerance). In particular, Fisher’s exact test was used for the frequency of small sample sets, the *t*-test was used for small sample sets with normal distribution, and the Mann-Whitney U test was used for small sample sets with non-normal distribution. The relative risk associated with an HLA allele/haplotype was estimated as an odds ratio (OR) with a 95% confidence interval (CI) from logistic regression. The ORs were determined using Haldane’s modification, which adds 0.5% to all of the cells in order to accommodate possible zero counts. Statistical significance was considered at a two-tailed *p*-value of <0.05 [27]. The Benjamini-Hochberg *p*-values of the multiple test were applied for the statistical analysis of the relationship between every single allele and the occurrence of MCARs in gout patients with multiple corrections for the number of HLA alleles tested [28].

## 3. Results

### 3.1. Characteristics of Subjects

This study included 50 patients with gout who were administered ALP at a dose of 150 mg/day. Among these 50 patients, 21 (42%) had ALP-induced MCARs, and 29 (58%) expressed ALP-tolerance. The demographic data are shown in Table 1. The ages of the patients ranged from 28 to 80 years, with an average age of 54 years for the patients with ALP-induced CAR, and 57 years for the ALP-tolerant patients. A total of 22 out of the 50 gout patients (44%) were over 60 years old: 6 of the 21 (28.6%) patients with ALP-induced MCARs and 16 of the 29 (55.2%) ALP-tolerant patients, respectively. The remaining 28 (56%) patients were under 60 years old: 15 of the 21 (71.4%) patients with ALP-induced MCARs and 13 of the 29 (44.8%) ALP-tolerant patients, respectively. There was only 1 female patient (2%), while the remaining 49 (98%) patients were male. Although the statistical results showed no significant difference, it implied that young patients were more sensitive to ALP than old patients. Other hematological parameters, biochemical parameters, and accompanying diseases in these patients were also examined (Table 1). The average concentrations of uric acid in the patients with ALP-induced MCARs and ALP-tolerant patients were 506 ± 18 µmol/L and 488 ± 21 µmol/L, respectively. Some patients also had other diseases, such as diabetes, dyslipidemia, hypertension and renal insufficiency. Among these, dyslipidemia was the only disease with the highest occurrence frequency, while the others were very rare (only one or no cases). In particular, 10 patients with ALP-induced MCARs and 15 with ALP-tolerant gout had dyslipidemia; however, there was no significant difference between these two groups. In addition, there were only 5 ALP-induced MCAR patients and 9 ALP-tolerant patients with no underlying disease; however, again, no significant difference was found between these two groups. More importantly, all 21 ALP-induced MCAR gout patients had mild skin lesions, including maculopapular rashes and itchiness (20 patients), while in the ALP-tolerant patients, 1 patient had itchiness and none had any skin lesions. In addition, although many hematological parameters were examined, only the eosinophil numbers were significantly increased in the ALP-induced MCAR gout patients compared to those in the ALP-tolerant gout patients (Table 1).

As shown in Table 2, the clinical investigations showed that 21 patients (42%) with ALP-induced ADRs had MCARs. All of the patients with ALP-induced MCARs had an onset of ADRs appearing within one month after exposure to ALP. In particular, 19 out of 21 (90.5%) patients had ADRs within two weeks of ALP administration. However, the symptoms did not become more severe with increased treatment time. 

### 3.2. Predominant HLA Alleles and Haplotypes in Patients with ALP-Induced MCARs

Because of the significant association between single nucleotide polymorphisms (SNPs) in the MHC region and ALP-induced CARs, two HLA-A and HLA-B loci of all 50 of the patients with gout were genotyped, and the data are shown in Table 2 and Table 3. The individual HLA alleles of the patients with ALP-induced MCARs were analyzed, and are presented in Table 2, in which highly frequent HLA alleles are highlighted in bold. In addition, the individual HLA alleles of the ALP-tolerant patients are shown in Table 3, in which highly frequent HLA alleles were found in the patients with ALP-induced MCARs but were rarely found in ALP-tolerant patients, and are highlighted in bold italic. 

The obtained data show that alleles HLA-B^*^58:01, HLA-A^*^24:02 and HLA-A^*^02:01 occurred with a higher frequency among patients with ALP-induced MCARs than among the ALP-tolerant patients. In particular, HLA-B^*^58:01 was present in eight out of 21 (38.1%) patients with ALP-induced MCARs, but in only 1 of the 29 (3.4%) patients in the ALP-tolerant group (odds ratio, 17.231; 95% CI, 1.947–152.496). 

In the 21 patients with ALP-induced MCARs, there were five single predominant alleles appearing—HLA-A^*^33:03, HLA-A^*^24:02, HLA-B^*^58:01, HLA-A^*^02:01 and HLA-A^*^29:01—with frequencies of 9/21 (42.9%), 10/21 (47.6%), 8/21 (38.1%), 8/21 (38.1%) and 5/21 (23.8%), respectively. However, except for HLA-B^*^58:01, the other four alleles (HLA-A^*^24:02, HLA-A^*^33:03, HLA-A^*^02:01, and HLA-A^*^29:01) were also predominant alleles occurring in ALP-tolerant patients, with frequencies of 10/29 (34.5%), 6/29 (20.7%), 6/29 (20.7%) and 6/29 (20.7%), respectively. Thus, there was no significant difference in the appearance frequencies of these four alleles, except for HLA-B^*^58:01, between the ALP-induced MCARs group and the ALP-tolerant group.

**Figure 1:**
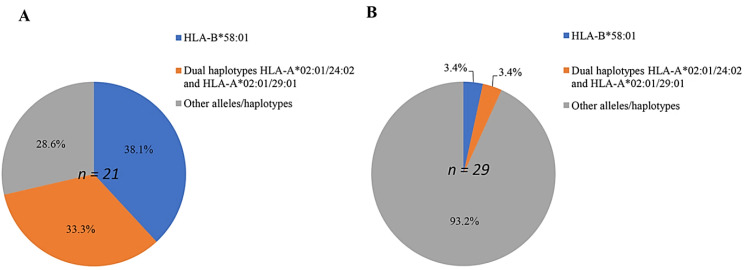
Percentage of predominant single alleles and dual haplotypes distributed in patients with ALP-induced MCARs (*n* = 21) (**A**), and in ALP-tolerant patients (*n* = 29) (**B**).

In order to investigate the role of individual alleles in patients with ALP-induced CARs, the odds ratios and *p*-values were calculated, and are presented in Table 4. Moreover, both HLA alleles and haplotypes, which have a significant relationship with ALP hypersensitivity syndrome in patients with gout compared with those in ALP-tolerant patients, are shown in Figure 1. The results demonstrated that the frequency of the single HLA-B^*^58:01 allele in Vietnamese patients with gout with ALP-induced MCARs was the highest (38.1%; 8 of 21) (Figure 1). 

This allele was significantly associated with MCARs occurrences in patients with an odds ratio of 17.231 (CI: 1.947–152.496). In contrast, other alleles had much lower odds ratios, with decreasing values of 2.875 (HLA-A^*^33:03) to 0.313 (HLA-A^*^01:01) for HLA-A alleles, and 2.947 (HLA-B^*^57:01) to 0.131 (HLA-B^*^46:01) for HLA-B alleles (Table 4). Nevertheless, it is interesting that the frequency of dual one-locus HLA-A^*^02:01/HLA-A^*^24:02 and HLA-A^*^02:01/HLA-A^*^29:01 in patients with ALP-induced MCARs was 7/21 (33.3%), while that in ALP-tolerant patients was only 1/29 (3.4%), with an odds ratio of 14.000 (95% CI: 1.565–125.262). 

Furthermore, in order to investigate the roles of single-allele and dual-allele haplotypes in ALP-induced MCARs patients with HLA-B^*^58:01 negative, the frequencies in ALP-induced MCARs patients and ALP-tolerant patients, as well as the odds ratios, were calculated, and are presented in Table 5. Among the 21 patients with ALP-induced MCARs, on the HLA-A locus, there were three single predominant alleles which occurred: HLA-A^*^24:02, HLA-A^*^02:01 and HLA-A^*^29:01, with frequencies of 6/21 (28.6%), 6/21 (28.6%) and 5/21 (23.8%), respectively. However, unfortunately, these three alleles of HLA-A^*^24:02, HLA-A^*^02:01 and HLA-A^*^29:01 were also predominant alleles occurring in ALP-tolerant patients, with frequencies of 10/29 (34.5%), 6/29 (20.7%), 6/29 (20.7%) and 6/29 (20.7%), respectively. The odds ratios for these three single alleles were quite low, at 0.760 (CI: 0.225–2.568), 1.533 (CI: 0.416–5.656) and 1.198 (CI: 0.311–4.609), respectively, with no significant difference. 

Interestingly, the odds ratios of dual one-locus HLA-A^*^02:01/HLA-A^*^24:02 and HLA-A^*^02:01/HLA-A^*^29:01, and both HLA-A^*^02:01/HLA-A^*^24:02 and HLA-A^*^02:01/HLA-A^*^29:01 were as high as 4.667 (CI: 0.450–48.411), 11.162 (CI: 0.545–228.662) and 11.200 (1.231–101.890), respectively, with significant differences to be recorded. More importantly, the appearance of dual one-locus HLA-A^*^02:01/HLA-A^*^24:02 and HLA-A^*^02:01/HLA-A^*^29:01 in patients with ALP-induced MCARs was accompanied by the absence of HLA-B^*^58:01. 

In addition, the obtained results demonstrated that the three single alleles, HLA-A^*^11:01, HLA-B^*^15:02 and HLA-B^*^46:01, were predominant in ALP-tolerant patients, with frequencies of 31% (9/29), 27.6% (8/29) and 27.6% (8/29), respectively, and the frequencies of these alleles in ALP-induced MCARs patients were all 4.8% (1/21); however, no significant difference was found. 

## 4. Discussion

It is well known that the presence of HLA-B^*^58:01 is closely related to the occurrence of severe adverse drug reactions (SJS, SJS/TEN, TEN, and DRESS) in the skin of patients (SCARs) when ALP is used for gout treatment, especially for Black and Asian people rather than white people [11,12,13,14,15,16,17,18,19,20,21,22,29]. Previous studies have found that the frequency of the HLA-B^*^58:01 allele in patients with ALP-induced SCARs in Han Chinese, Taiwanese, Thai, Korean, Japanese and Vietnamese populations varies between 90% and 100%, while the frequency of the HLA-B^*^58:01 allele in healthy people is 6–10%, depending on the ethnic population [11,12,13,14,15,16,17,18,19,20,21,22]. Therefore, the HLA-B^*^58:01 allele has been approved as a diagnostic marker for ALP-induced SCARs, and its screening is recommended before using ALP for gout treatment [3,4]. However, to date, the relationship between the presence of the HLA-B^*^58:01 allele and the occurrence of MCARs has not been thoroughly investigated. A previous study in a Han Chinese population showed that 100% of ALP-treated patients with gout and MCARs carry the HLA-B^*^58:01 allele [12]. However, another study on Kinh Vietnamese reported that only approximately 6.8% of a total of 75 patients with gout and MCARs were positive for the HLA-B^*^58:01 allele [20], a frequency that is almost equal to that observed in healthy Kinh Vietnamese individuals (6.5%). In this study, we found that the frequency of the HLA-B^*^58:01 allele in Vietnamese patients with ALP-induced MCARs was 38.1%. The frequency of the HLA-B^*^58:01 allele in Vietnamese patients with ALP-induced MCARs varied in different studies (the current study and [20]), which may be due to the limited number of patients enrolled in the studies or the differences in the subject classifications between the two studies. These results suggest that further studies should be conducted in order to address this association, with an increased number of patients and with a focus on other ethnic groups.

Moreover, we also revealed that besides the HLA-B^*^58:01 allele, several other alleles, such as HLA-A^*^33:03, HLA-A^*^24:02, HLA-B^*^58:01, HLA-A^*^02:01 and HLA-A^*^29:01, also appeared in patients with gout at a high frequency. This result is in accordance with previous reports [17,26,30,31]. However, we found that there was no significant difference in the frequency of these alleles in patients with ALP-induced MCARs compared with those in ALP-tolerant patients, as represented by the odds ratios shown in Table 4. 

More importantly, we demonstrated that dual haplotypes on one locus (HLA-A locus), including HLA-A^*^02:01/HLA-A^*^24:02 and HLA-A^*^02:01/HLA-A^*^29:01, appeared in patients with ALP-induced MCARs at significantly higher frequencies than those in the ALP-tolerant patients. Interestingly, the occurrence of these dual haplotypes was independent of the appearance of the HLA-B^*^58:01 allele (Table 5). In summary, the percentage of patients with ALP-induced MCARs who carried both the single HLA-B^*^58:01 allele and dual one-locus haplotypes of HLA-A^*^02:01/HLA-A^*^24:02 and HLA-A^*^02:01/HLA-A^*^29:01 was approximately 70%, while that in ALP-tolerant patients was only approximately 6.7%. 

In addition, this study also revealed that HLA-A^*^11:01, HLA-B^*^15:02 and HLA-B^*^46:01 alleles appeared in ALP-tolerant patients with higher frequencies compared with those in patients with ALP-induced MCARs (Table 5). A previous study showed that the general frequencies of HLA-A^*^11:01, HLA-B^*^15:02 and HLA-B^*^46:01 in Kinh Vietnamese patients were approximately 22%, 14% and 12%, respectively [19]. Other studies also reported that HLA-A^*^11:01, HLA-B^*^15:02, and HLA-B^*^46:01 were present in patients with ALP-induced SCARs at low frequencies (<6%) [32]. These results suggest that gout patients carrying HLA-A^*^11:01, HLA-B^*^15:02 and HLA-B^*^46:01 alleles may have a lower risk of allopurinol hypersensitivity.

## 5. Conclusions

This study is the first to demonstrate the role of HLA-allele polymorphisms in patients with gout and MCARs in the Vietnamese population. Based on the results obtained, we suggest that although the HLA-B^*^58:01 allele may be a predominant allele in patients with ALP-induced CARs, the correlation between the presence of the HLA-B^*^58:01 allele and the occurrence of MCARs in ALP-treated patients with gout is not as strong as that of SCARs. In addition to the presence of the HLA-B^*^58:01 allele, one-locus haplotypes of HLA-A^*^02:01/HLA-A^*^24:02 and HLA-A^*^02:01/HLA-A^*^29:01 probably play an important role in the occurrence of ADRs with mild symptoms in patients with gout treated with ALP. These data may be useful for consulting doctors before the prescription of ALP for gout treatment, in order to reduce the risk of not only SCARs but also MCARs. 

## Figures and Tables

**Table 1:** Demographic data of the patients with gout, with and without cutaneous adverse reactions (MCARs).

Characteristic	MCARs Cases (N = 21)	Tolerant Cases (N = 29)	*p*-Value
Starting dose (mg/day)	150	150	
**Age (year)**	Average: 54	Average: 57	
Min-Max: 28–75	>60 years: 06;	>60 years: 16;	>0.05 *
>60 years: 22; ≤60 years: 28	≤60 years: 15	≤60 years: 13	
**Gender**			
Male	20	29	0.420
Female	1	0	
**Underlying disease**			
Diabetes	1	0	0.420 **
Dyslipidemia	10	15	1.000
Hypertension	1	0	
Liver disease	0	1	0.420 **
Diabetes/Chronic renal insufficiency	1	0	1.000 **
Diabetes/Dyslipidemia	1	1	0.420 **
Diabetes/Hypertension	1	0	1.000 **
Dyslipidemia/Hypertension	1	0	0.420 **
Dyslipidemia/Liver disease	0	1	1.000 **
Dyslipidemia/Hypertension/Liver disease	0	2	0.420 **
None	5	9	0.420 **
**Skin lesions**			
Maculopapular rashes	20	0	<0.001 **
Itchy	1	0	0.420 **
Uric concentration (µmol/L)	506.00 ± 18.31	488.28 ± 20.65	0.542 *
ALT (IU/mL)	40.29 ± 6.22	45.48 ± 6.61	0.461 ^†^
AST (IU/mL)	48.81 ± 8.75	40.59 ± 6.03	0.330 ^†^
Creatinine (mmol/L)	101.38 ± 7.14	95.42 ± 6.31	0.687 ^†^
BUN (mg/mL)	6.29 ± 0.60	6.06 ± 0.43	0.945 ^†^
WBC count (G/L)	11.72 ± 0.61	16.05 ± 4.56	0.969 ^†^
RBC count (T/L)	4.33 ± 0.14	4.25 ± 0.13	0.657 *
Platelet count (G/L)	344.43 ± 31.69	351.14 ± 26.12	0.623 ^†^
Hemoglobin count (G/L)	130.24 ± 4.47	125.72 ± 4.04	0.293 ^†^
Eosinophil (%)	2.82 ± 0.44	1.49 ± 0.43	0.004 ^†^
Glucose (mg/L)	6.33 ± 0.35	6.29 ± 0.24	0.629 ^†^
CRP (mg/L)	5.98 ± 1.63	8.25 ± 1.71	0.665 ^†^
Cholesterol (mmol/L)	5.25 ± 0.28	5.60 ± 0.26	0.701 ^†^
Triglyceride (mmol/L)	2.68 ± 0.23	2.89 ± 0.24	0.537 *

ALT, alanine aminotransferase; AST, aspartate transaminase; IU, international units; BUN, blood urea nitrogen; WBC, white blood cell; RBC, red blood cell; CRP, C-reactive protein. All of the values are presented as the mean ± SE or n. * T-test was used; ^†^ Mann-Whitney U was used; ** Fisher’s exact test was used.

**Table 2:** Patients with ALP-induced MCAR gout with the onset time of ADR and the corresponding genotypes of the HLA alleles.

No.	Age/Sex	Time of Day with MCARs	Genotype of HLA	
			Locus A	Locus B
1	75/Male	7	24:02/24:02	52:01/52:01
2	46/Male	5	24:02/33:03	40:01/58:01
3	45/Male	6	24:02/33:03	15:02/58:01
4	29/Male	32	11:01/33:03	38:02/58:01
5	43/Male	5	24:02/29:01	07:02/15:02
6	30/Male	10	11:01/33:03	38:02/58:01
7	74/Male	10	01:01/02:03	46:01/57:01
8	39/Male	9	02:01/24:02	13:01/38:02
9	61/Male	20	02:01/24:02	15:12/57:01
10	73/Male	2	24:02/29:01	07:05/15:25
11	71/Female	7	11:01/33:03	40:02/58:01
12	28/Male	2	02:01/33:03	46:01/58:01
13	55/Male	5	02:01/29:01	07:05/40:01
14	59/Male	3	02:01/24:02	13:01/38:02
15	55/Male	5	02:01/29:01	07:05/40:01
16	58/Male	5	11:01/33:03	15:25/44:03
17	54/Male	14	02:01/29:01	07:05/40:01
18	57/Male	8	02:03/24:02	56:04/58:01
19	53/Male	5	02:01/24:02	13:01/58:01
20	70/Male	7	26:01/33:03	27:04/44:03
21	53/Male	6	11:02/33:03	15:12/40:01

**Table 3:** ALP-tolerant patients with gout, with the corresponding genotypes of HLA alleles.

No.	Age/Sex	Locus A	Locus B
1	54/Male	11:01/33:03	15:01/38:02
2	28/Male	11:01/33:03	13:01/44:03
3	47/Male	11:02/24:02	39:01/40:01
4	65/Male	24:07/29:01	07:02/07:05
5	68/Male	11:02/24:02	07:05/15:12
6	28/Male	29:01/33:03	07:05/44:03
7	53/Male	01:01/68:01	07:02/37:01
8	57/Male	24:02/29:01	07:05/13:01
9	80/Male	02:03/03:01	27:05/40:01
10	68/Male	01:01/33:03	57:01/58:01
11	66/Male	02:01/24:03	15:12/46:01
12	69/Male	24:02/29:01	07:05/38:02
13	32/Male	11:01/24:02	15:02/15:12
14	46/Male	02:06/11:01	15:02/15:02
15	64/Male	24:02/33:03	15:02/46:01
16	61/Male	01:01/02:01	15:02/46:01
17	45/Male	02:03/11:01	15:01/15:02
18	66/Male	02:03/29:01	07:05/38:02
19	41/Male	02:06/11:01	15:02/40:01
20	68/Male	01:01/11:01	37:01/38:02
21	61/Male	24:02/24:02	40:01/46:01
22	56/Male	02:03/29:01	07:05/13:01
23	62/Male	02:01/11:02	15:02/46:01
24	59/Male	02:01/31:01	07:05/46:01
25	52/Male	11:01/11:01	15:02/35:05
26	67/Male	02:01/24:02	46:01/51:01
27	67/Male	11:01/24:02	51:01/54:01
28	62/Male	24:02/33:03	18:01/27:04
29	60/Male	02:01/02:01	46:01/46:01

**Table 4:** Summary of cases with different single allele frequencies in patients with ALP-induced MCARs and ALP-tolerant patients.

No.	HLA Allele	ALP-Induced MCARs Cases (N = 21)	ALP Tolerant Cases (N = 29)	Odds Ratio (95% CI)	*p*-Value
	HLA-A				
1	33:03	9	6	2.875 (0.826–10.001)	0.884 ^Φ^
2	02:01	8	6	2.359 (0.670–8.301)	0.884 ^Φ^
3	24:02	10	10	1.727 (0.548–5.448)	0.884 ^Φ^
4	26:01	1	1	1.400 (0.083–23.737)	1 ^Φ^
5	29:01	5	6	1.198 (0.311–4.609)	1 ^Φ^
6	02:03	2	4	0.658 (0.109–3.977)	0.892 ^Φ^
7	11:01	4	9	0.523 (0.136–2.004)	0.892 ^Φ^
8	11:02	1	3	0.433 (0.042–4.485)	0.892 ^Φ^
9	01:01	1	4	0.313 (0.032–3.021)	0.884 ^Φ^
	Dual HLA-A				
1	02:01/24:02	4	1	6.5882 (0.679–63.942)	0.148 *
2	02:01/29:01	3	0 *^a^*	11.162 (0.545–228.662)	0.027
3	02:01/24:02 or 02:01/29:01	7	1	14.000 (1.565–125.262)	0.007
	HLA-B				
1	58:01	8	1	17.231 (1.947–152.496)	0.03 ^Φ^
2	15:25	2	0 *^a^*	7.564 (0.344–166.209)	0.949 ^Φ^
3	40:02	1	0 *^a^*	4.317 (0.167–111.328)	1 ^Φ^
4	52:01	1	0 *^a^*	4.317 (0.167–111.328)	1 ^Φ^
5	57:01	2	1	2.947 (0.249–34.850)	1 ^Φ^
6	13:01	3	3	1.444 (0.261–7.982)	1 ^Φ^
7	40:01	5	4	1.953 (0.455–8.364)	1 ^Φ^
8	38:02	4	4	1.471 (0.323–6.702)	1 ^Φ^
9	15:12	2	3	0.912 (0.139–6.005)	1 ^Φ^
10	44:03	2	2	1.421 (0.184–10.994)	1 ^Φ^
11	27:04	1	1	1.400 (0.083–23.737)	1 ^Φ^
12	07:05	4	8	0.618 (0.159–2.406)	1 ^Φ^
13	07:02	1	2	0.675 (0.057–7.973)	1 ^Φ^
14	15:02	2	8	0.276 (0.052–1.467)	0.8 ^Φ^
15	46:01	2	8	0.276 (0.052–1.467)	0.8 ^Φ^

*^a^* As zero is present in Table 5, 0.5 was added to all of the cells before calculating the odds ratios. * Fisher’s exact test was used; **^Φ^** the Benjamini Hochberg *p*-value of multiple tests was indicated.

**Table 5:** Allele frequencies in ALP-induced MCARs patients and ALP-tolerant patients with HLA-B^*^58:01 negative.

No.	HLA Allele	ALP-Induced MCARs Cases (N = 21)	ALP Tolerant Cases (N = 29)	Odds Ratio (95% CI)	*p*-Value
	HLA-A				
1	02:01	6	6	1.533 (0.416–5.656)	1 ^Φ^
2	26:01	1	1	1.400 (0.083–23.737)	1 ^Φ^
3	29:01	5	6	1.198 (0.311–4.609)	1 ^Φ^
4	33:03	3	5	0.800 (0.169–3.793)	1 ^Φ^
5	24:02	6	10	0.760 (0.225–2.568)	1 ^Φ^
6	01:01	1	3	0.444 (0.042–4.485)	1 ^Φ^
7	11:02	1	3	0.433 (0.041–4.485)	1 ^Φ^
8	02:03	1	4	0.313 (0.032–3.021)	1 ^Φ^
9	11:01	1	9	0.111 (0.013–0.961)	0.279 ^Φ^
	Dual HLA-A				
1	02:01/24:02	3	1	4.667 (0.450–48.411)	0.297 *
2	02:01/29:01	3	0 *^a^*	11.162 (0.545–228.662)	0.027 *
3	02:01/24:02 or 02:01/29:01	6	1	11.200 (1.231–101.890)	0.033 *
	HLA-B				
1	15:25	2	0 *^a^*	7.564 (0.344–166.209)	0.822 ^Φ^
2	57:01	2	0 *^a^*	7.564 (0.344–166.209)	0.822 ^Φ^
3	52:01	1	0 *^a^*	4.317 (0.167–111.328)	1 ^Φ^
4	40:01	4	4	1.471 (0.323–6.702)	1 ^Φ^
5	44:03	2	2	1.421 (0.184–10.994)	1 ^Φ^
6	27:04	1	1	1.400 (0.083–23.737)	1 ^Φ^
7	13:01	2	3	0.912 (0.139–6.005)	1 ^Φ^
8	15:12	2	3	0.912 (0.139–6.005)	1 ^Φ^
9	07:02	1	2	0.675 (0.057–7.973)	1 ^Φ^
10	38:02	2	4	0.658 (0.109–3.977)	1 ^Φ^
11	07:05	4	8	0.618 (0.159–2.406)	1 ^Φ^
12	15:02	1	8	0.131 (0.015–1.146)	0.397 ^Φ^
13	46:01	1	8	0.131 (0.015–1.146)	0.397 ^Φ^

*^a^* As zero is present in the table, 0.5 was added to all of the cells before calculating the odds ratios. * Fisher’s exact test was used; **^Φ^** the Benjamini Hochberg *p*-values of multiple tests were indicated.

## Data Availability

The data presented in this study are available on request from the corresponding author.
